# Domestication of the novel alcohologenic acetogen *Clostridium* sp. AWRP: from isolation to characterization for syngas fermentation

**DOI:** 10.1186/s13068-019-1570-0

**Published:** 2019-09-23

**Authors:** Joungmin Lee, Jin Woo Lee, Cheol Gi Chae, Soo Jae Kwon, Yun Jae Kim, Jung-Hyun Lee, Hyun Sook Lee

**Affiliations:** 1 Marine Biotechnology Research Center, Korea Institute of Ocean Science and Technology, Haeyangro 385, Busan, 49111 Republic of Korea; 2 Department of Marine Biotechnology, University of Science and Technology, Daejeon, Republic of Korea

**Keywords:** Acetogens, Syngas fermentation, Wood–Ljungdahl pathway, Ethanol, *Clostridium*

## Abstract

**Background:**

Gas-fermenting acetogens have received a great deal of attention for their ability to grow on various syngas and waste gas containing carbon monoxide (CO), producing acetate as the primary metabolite. Among them, some *Clostridium* species, such as *C. ljungdahlii* and *C. autoethanogenum*, are of particular interest as they produce fuel alcohols as well. Despite recent efforts, alcohol production by these species is still unsatisfactory due to their low productivity and acetate accumulation, necessitating the isolation of strains with better phenotypes.

**Results:**

In this study, a novel alcohol-producing acetogen (*Clostridium* sp. AWRP) was isolated, and its complete genome was sequenced. This bacterium belongs the same phylogenetic group as *C. ljungdahlii*, *C. autoethanogenum*, *C. ragsdalei*, and *C. coskatii* based on 16S rRNA homology; however, the levels of genome-wide average nucleotide identity (gANI) for strain AWRP compared with these strains range between 95 and 96%, suggesting that this strain can be classified as a novel species. In addition, strain AWRP produced a substantial amount of ethanol (70–90 mM) from syngas in batch serum bottle cultures, which was comparable to or even exceeded the typical values obtained using its close relatives cultivated under similar conditions. In a batch bioreactor, strain AWRP produced 119 and 12 mM of ethanol and 2,3-butanediol, respectively, while yielding only 1.4 mM of residual acetate. Interestingly, the alcohologenesis of this strain was strongly affected by oxidoreduction potential (ORP), which has not been reported with other gas-fermenting clostridia.

**Conclusion:**

Considering its ethanol production under low oxidoreduction potential (ORP) conditions, *Clostridium* sp. AWRP will be an interesting host for biochemical studies to understand the physiology of alcohol-producing acetogens, which will contribute to metabolic engineering of those strains for the production of alcohols and other value-added compounds from syngas.

## Background

Global warming has become a great threat to the sustainability of humanity, as stated in the Paris Agreement adopted at the 21st meeting of the Conference of the Parties. CO_2_ emission generated by the use of fossil fuels is a major cause of global warming, and a shift toward the production of value-added chemicals using renewable resources is urgently needed [1]. Beyond the fermentation of renewable biomass, the biological conversion of gaseous feedstocks (CO, CO_2_, and H_2_) has attracted considerable attention [2]. These substrates can be obtained via the incomplete combustion of inexpensive materials, such as inedible biomass, waste, or coal [3, 4]. Among the microorganisms that assimilate gaseous substrates, acetogens are of interest because they are able to utilize either CO or CO_2_ plus H_2_ [5]. Acetogens employ the Wood–Ljungdahl (WL) pathway (also known as reductive acetyl-CoA pathway) to assimilate CO_2_ into acetyl-CoA, with acetate being the major product [6, 7]. Since CO is a precursor for the synthesis of acetyl-CoA in this pathway, several acetogens can utilize CO as a sole carbon source [7, 8].

Acetogens belonging to the genus *Clostridium* have been increasingly studied, since some of these species are able to produce several alcohols such as ethanol, butanol, and 2,3-butanediol in addition to acetate [3, 9–12]. It was previously reported that a large amount of ethanol (48 g L^−1^) could be produced by continuous fermentation of coal synthesis gas by *C. ljungdahlii* [3]. Other potential alcohol producers include *C. autoethanogenum*, *C. ragsdalei*, and *C. carboxidivorans* [5, 13–15]. In addition to biochemical studies [16, 17], a great deal of effort has been focused on systems biology [18–20] and metabolic engineering [14, 21] of these bacteria over the last decade. Nevertheless, it may take a substantial amount of time to improve their performances solely through metabolic engineering due to our limited knowledge [22]. For example, a recent study described the genes associated with ethanol production in *C. autoethanogenum*, but the inactivation of these genes often caused a prolonged lag phase during autotrophic growth [14]. Thus, it remains necessary to isolate novel strains that have more desirable traits (e.g., a higher gas consumption rate, ethanol selectivity, tolerance to fermentation products, etc.), to exploit them for the production of useful chemicals and to better understand the physiology of gas-fermenting microorganisms.

Here, we report a novel alcohol-producing acetogen, *Clostridium* sp. AWRP, isolated from a wetland in Ansan, South Korea, and its complete genome sequence. Based on its 16S ribosomal RNA (rRNA) gene sequence, this strain belongs to the same clade with *C. ljungdahlii*, *C. autoethanogenum*, *C. ragsdalei*, and *C. coskatii*. We also examined its cultural characteristics during autotrophic growth, which showed that strain AWRP was able to produce ethanol with high selectivity. In a bioreactor experiment, strain AWRP produced ethanol with a high selectivity under low-ORP conditions, which has not been reported in other alcohol-producing acetogens.

## Results

### Isolation and identification of *Clostridium* sp. AWRP

Sediments and livestock sludge samples were collected from marine sediments, rice paddies, lakes, wetlands, poultry and cattle farms. Enrichment culture techniques were employed using the AM medium to obtain autotrophic microorganisms capable of utilizing a synthetic blend syngas (50% CO, 10% H_2_, 10% CO_2_, and 30% N_2_). After the enrichment culture, thirty-seven isolates were obtained from different samples and then grown individually to screen for CO utilization. One isolate, AWRP, which showed rapid CO consumption and a short lag phase was chosen for subsequent identification. The AWRP strain is mesophilic and slightly acidophilic: the optimum growth temperature and pH were 37 °C and 6.0–6.5, respectively.

**Fig. 1:**
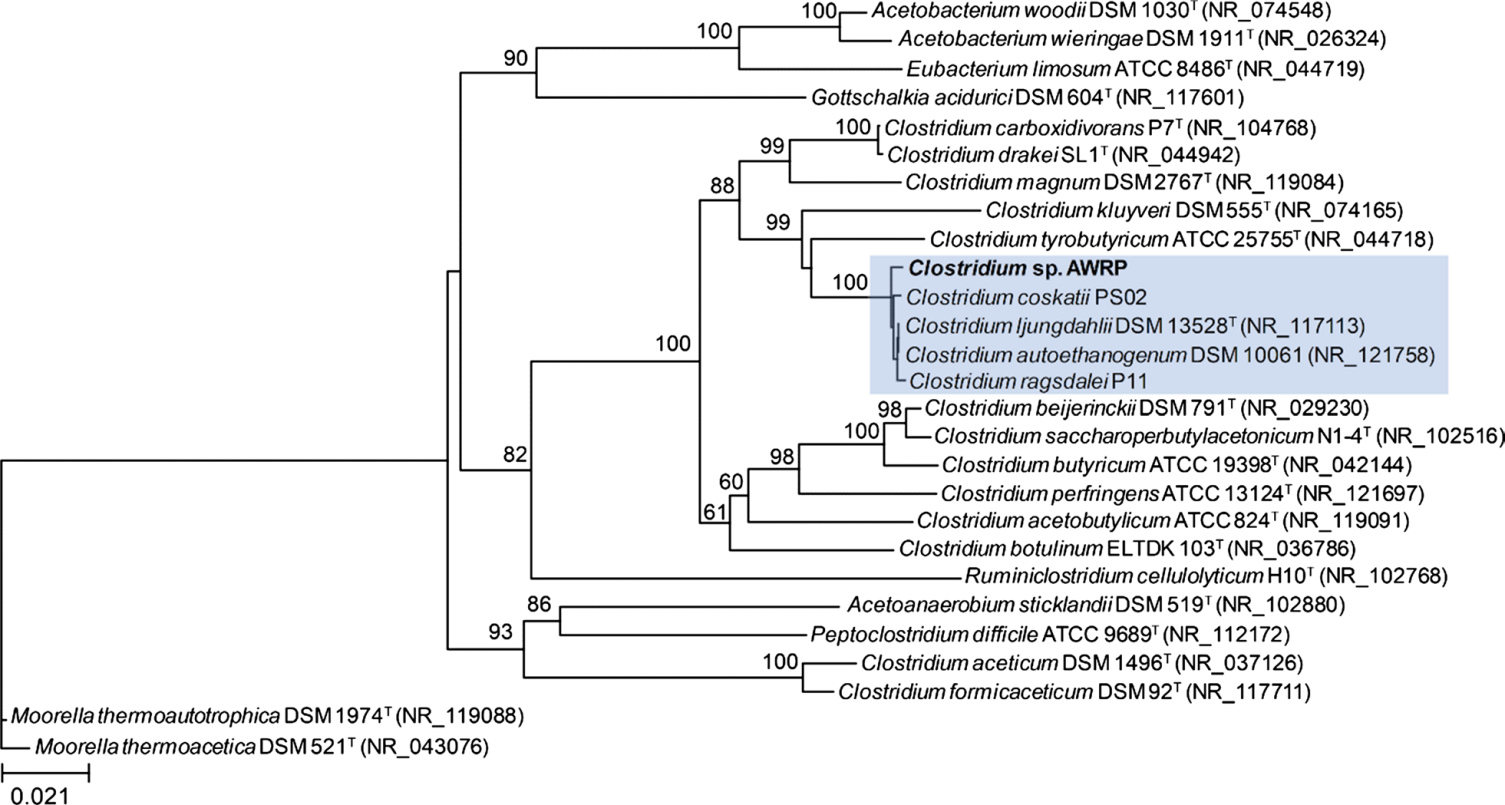
16S rRNA phylogenetic tree of several acetogens and other *Clostridium* species. *Moorella thermoacetica* DSM 521 was chosen as the outgroup. The blue shaded box shows the clade that includes *Clostridium* sp. AWRP and its close relatives. The number shown above each node represents the bootstrap values (expressed as percentages of 1000 resampled datasets) and is only indicated for the values greater than 60%

The isolate was subjected to 16S rRNA gene sequencing and was shown to belong to the genus *Clostridium*. After whole genome sequencing of *Clostridium* sp. AWRP, its 16S rRNA sequence was compared to those of other clostridial species: strain AWRP was clustered in a monophyletic group together with *C. ljungdahlii*, *C. autoethanogenum*, *C. ragsdalei*, and *C. coskatii* (100% bootstrap value; Fig. 1). To distinguish these closely related species, we performed a genome-wide average nucleotide identity (gANI) analysis (Table 1; see the next section for details of its genome) [23]. Pairwise gANI values between strain AWRP and the others range between 95 and 96%, slightly below an ANI-based cutoff (96.5%) for species delineation [23]. The gANI value of strain AWRP is highest with *C. ragsdalei* (95.5%), but the alignment fraction was only 52%, which is less than the minimum threshold (60%) for this method [23].

**Table 1:** Average nucleotide identity (ANI) values of AWRP and its close relatives

ANI value^a^ (%)	Query				
	*C. ljungdahlii*	*C. autoethanogenum*	*C. coskatii*	*C. ragsdalei*	AWRP
Subject					
*C. ljungdahlii*	–	99.3 (89.9)	98.3 (84.5)	95.9 (66.8)	95.1 (75.8)
*C. autoethanogenum*	–	–	98.1 (79.9)	95.9 (66.0)	95.2 (72.3)
*C. coskatii*	–	–	–	95.8 (65.6)	95.1 (74.7)
*C. ragsdalei*	–	–	–	–	95.5 (52.1)
AWRP	–	–	–	–	–

^a^Values in the parentheses are alignment fractions in percentage

### Features of the *Clostridium* sp. AWRP genome

**Fig. 2:**
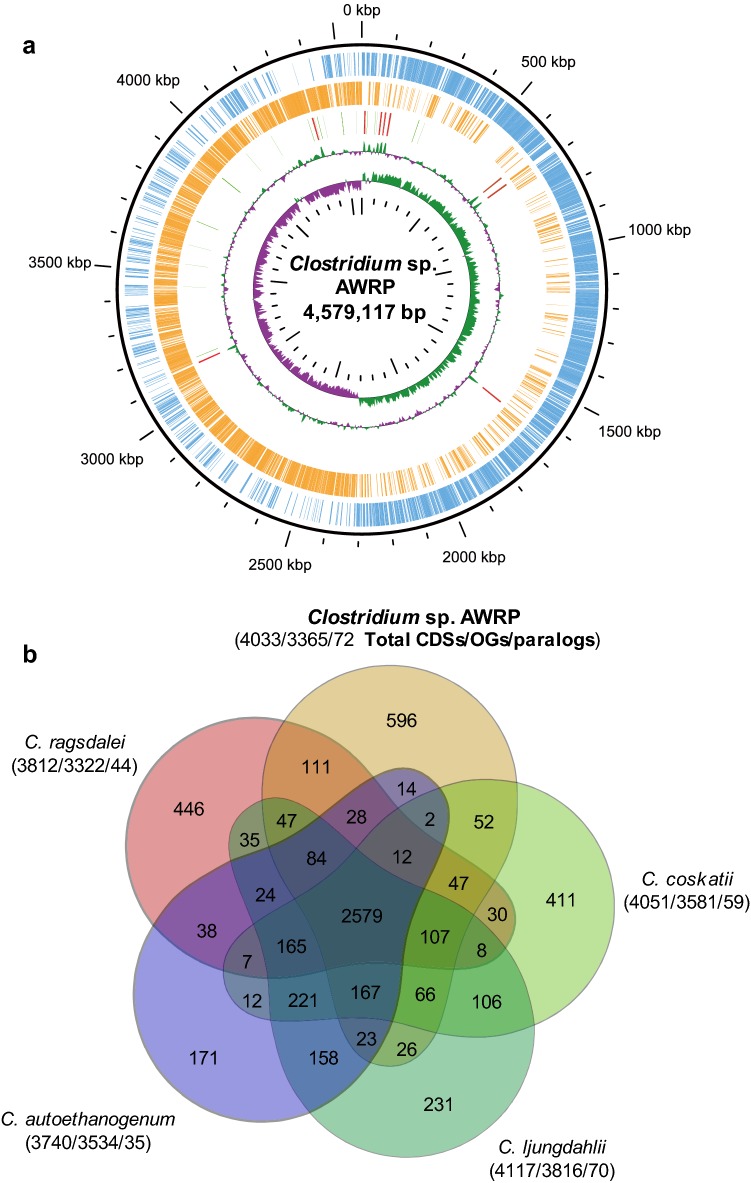
**a** Representation of the *Clostridium* sp. AWRP chromosome. The outermost ring shows the coordinates. The coding sequences in the sense and antisense strands are colored blue and orange, respectively. tRNAs and rRNAs are shown in the third ring and colored red and green, respectively. The fourth and fifth rings represent the deviations in the GC content and skew from their average values, respectively (purple, negative deviation; and dark green, positive deviation). **b** Venn diagram showing the numbers of orthologous genes (OGs) in the genomes of *Clostridium* sp. AWRP and its relatives. Detection of the orthologs was performed according to Bengelsdorf et al. [29], after excluding pseudogenes. The number of total protein-coding genes, OGs and paralogs are shown under the species names. Note that the numbers of total protein-coding genes for the four reported strains have been changed from the previous work, due to the recent reannotation of prokaryotic genomes by NCBI

The complete genome of *Clostridium* sp. AWRP consists of one 4.58 Mbp circular chromosome with a GC content of 31.2% (Fig. 2a). The chromosome encodes 4033 protein-coding, 27 tRNA, and 72 rRNA genes. From the orthology analysis against all protein-coding genes of strain AWRP and the four close strains from the same phylogenetic group (see Fig. 1), the AWRP genome was shown to harbor 596 singletons (i.e., strain-specific genes) of 3691 orthologous genes, the highest number among the five strains (Fig. 2b). The genome harbors eight genes encoding the LtrA-like reverse transcriptase of mobile group II introns, orthologs of which were not identified in those of the other strains. The CRISPR system of strain AWRP is presumed to be dysfunctional, as the genome harbors only one gene encoding a Cas2 protein (DMR38_05220), although three CRISPR arrays are predicted. The genome consists of three putative prophage regions (DMR38_09360-DMR38_09475, DMR38_15615-DMR38_15715, and DMR38_20795-DMR38_20915).

**Fig. 3:**
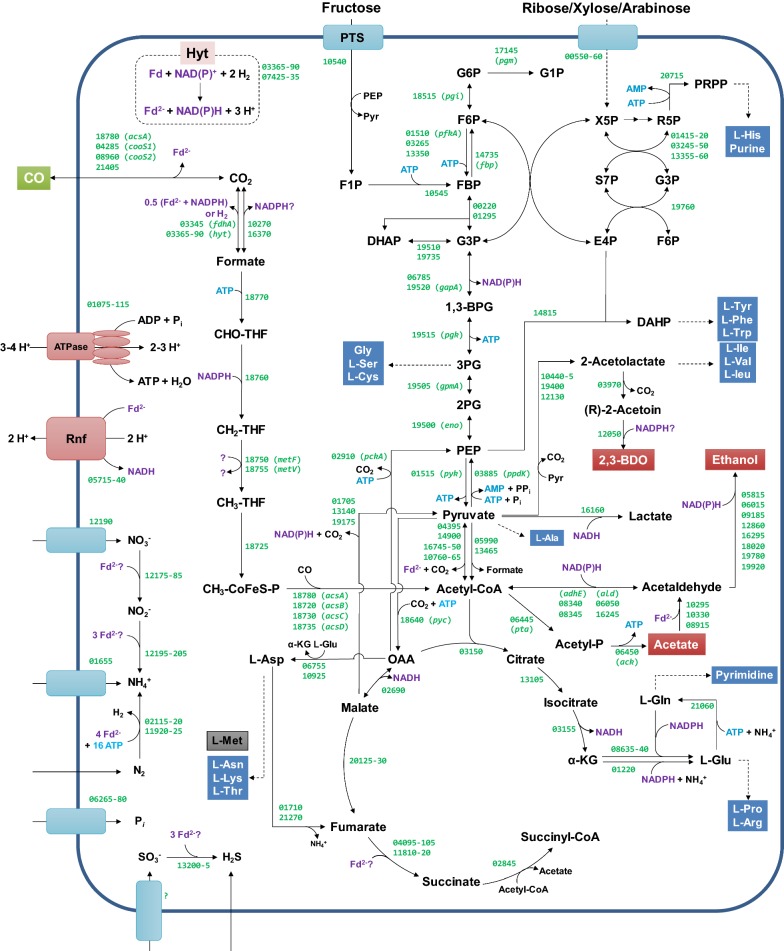
Reconstruction of *Clostridium* sp. AWRP central metabolism based on the genomic information. Locus numbers of the corresponding genes are shown in green on each reaction without the locus prefix. A lumped reaction or pathway is shown in a dashed arrow for simplicity; the gray box indicates l-methionine auxotrophy of strain AWRP. It was assumed that three to four protons are required to phosphorylate one ATP according to a previous study on *Clostridium paradoxum* [54], and that two protons are translocated per ferredoxin oxidized [8, 55]. The electron donor of the methylenetetrahydrofolate reductase is indicated with question marks since it is still unclear in clostridial species [17]

The central metabolism of strain AWRP is summarized in Fig. 3 based on its genome annotation. All genes encoding the enzymes for methyl and carbonyl branches of the WL pathway are present in the AWRP genome. In addition to *acsA1* (DMR38_18780), encoding nickel-dependent anaerobic CO dehydrogenase (CODH) in the acetyl-CoA synthase complex, three additional copies of CODH genes are present in the genome. Two of these genes (DMR38_04285 and DMR38_08960) are orthologs of *cooS1* and *cooS2* in *C. autoethanogenum*, respectively [22], while the other one (DMR38_21405) appears to be a paralog of *acsA*, which is not present in the genomes of *C. ljungdahlii* and *C. autoethanogenum* but is harbored by *C. ragsdalei*. As in *C. autoethanogenum* and *C. ljungdahlii*, there is a large, single cluster containing genes for both branches of the WL pathway (DMR38_18710-DMR38_18780), except for those encoding a formate dehydrogenase (FDH) complex. The genome harbors three genes encoding FDH, two of which (DMR38_03345 and DMR38_10270) are selenocysteine containing while the other one (DMR38_16370) is not.

All acetogenic bacteria are known to require additional energy-conserving reactions during autotrophic growth, as the WL pathway itself does not yield net ATP gain by substrate-level phosphorylation [8]. The AWRP genome contains genes encoding the Rnf complex (DMR38_05715-DMR38_05740) and ATP synthase (DMR38_01075-DMR38_01110) but not those encoding Ech-type hydrogenases present in *Moorella* species. In addition, the electron-bifurcating transhydrogenase NfnAB, which was first identified in *C. kluyveri*, is encoded by a single gene in the genome (DMR38_18560). The genome harbors genes encoding five iron-only hydrogenases (DMR38_07425-07435, DMR38_03365-03390, DMR38_08575-08585, DMR38_10370, and DMR38_18550) and one nickel–iron hydrogenase (DMR38_14570-DMR38-14575). The putative hydrogenase encoded by the first cluster has similarities with the trimeric, electron-bifurcating hydrogenase of *Thermotoga maritima* [24]. The second one is adjacent to a gene cluster encoding FDH, and it is likely to form a H_2_-dependent FDH complex as reported in *C. autoethanogenum* [16].

The AWRP genome harbors an operon encoding phosphotransacetylase and acetate kinase (DMR38_06445-DMR38_06450; Fig. 3). The genome harbors three genes encoding aldehyde:ferredoxin oxidoreductase (AOR), two of which (DMR38_10295 and DMR38_10330) are flanking an operon encoding a tungstate ABC transporter. The third AOR gene (DMR38_08915) is distantly located from the other AOR genes. Several genes encoding aldehyde and alcohol dehydrogenases were identified, some of which could be involved in ethanol production in this strain. Strain AWRP also contains the genes for 2,3-butanediol synthesis, including acetolactate synthase (DMR38_10440-DMR38_10445, DMR38_19400, and DMR38_12130), acetolactate decarboxylase (DMR38_03970), and 2,3-butanediol dehydrogenase (DMR38_12050).

The AWRP genome harbors the genes for nitrogen assimilation, including nitrogenase (DMR38_02115-DMR38_02120 and DMR38_11920-DMR38_11925) and an ammonium transporter (DMR38_01655), respectively. Also, the strain AWRP may assimilate nitrate or nitrite using nitrate/nitrite transporter and nitrate reductase (encoded by DMR38_12175-DMR38_12190). No genes annotated as nitrite reductase were present in the genome. The genes encoding sulfite reductase (DMR38_12195-DMR38_12205) are located adjacent to the nitrate reductase genes, suggesting that the products of these genes are presumably involved in nitrite reduction [25]. However, the AWRP strain may require several organic nitrogen sources for growth as the biosynthetic pathways for the following compounds are incomplete: l-methionine (due to absence of cystathionine β-lyase), thiamine, biotin, pyridoxal, pantothenate, and 4-aminobenzoate.

### Metabolite profiles of *Clostridium* sp. AWRP under autotrophic conditions

Since the genome of *Clostridium* sp. AWRP was observed to be distinct from those of its close relatives, we next characterized its metabolite profiles under autotrophic growth conditions. Prior to the characterization, we first attempted to set a culture protocol by cultivating strain AWRP in serum bottles containing different volumes of the PETC medium: volumetric gas-to-liquid ratios were ca. 14.8, 6.9, and 3.0 for 10, 20, and 40 mL of the culture volume, respectively (Table 2). The results indicated that supplying high amounts of gaseous substrates was necessary to observe alcohol production, where the ethanol and 2,3-butanediol titers were highest with a 10 mL culture volume (94 and 6 mM, respectively), and they decreased as the culture volume increased (Table 2). In the 40-mL cultures, the ethanol titer was only 8 mM, much lower than acetate titer (28 mM; Table 2). Based on this result, we performed serum bottle cultures with 10 mL culture volume in subsequent cultures.

**Table 2:** Culture profiles of *Clostridium* sp. AWRP in the PETC medium with various gas/liquid ratios

Parameters	Gas-to-liquid ratio (relative)		
	3.0 (1)	6.9 (2.34)	14.8 (5.02)
Gas^a^ (mM)			
CO consumed	157 ± 1	368 ± 2	778 ± 7
H_2_ consumed	29 ± 2	15 ± 5	7 ± 3
CO_2_ produced	52 ± 2	186 ± 11	434 ± 3
Metabolite (mM)			
Acetate	28 ± 2	40 ± 4	14 ± 2
Ethanol	8 ± 1	24 ± 2	94 ± 0
2,3-Butanediol	ND^b^	2 ± 0	6 ± 1
C-recovery^c^ (%)	79 ± 1	87 ± 2	87 ± 0

^a^Normalized to the culture volume by: Δ(Headspace gas concentration) × (headspace volume)/(culture volume)

^b^Not detected

^c^Carbon recovery; cell mass was not included

**Fig. 4:**
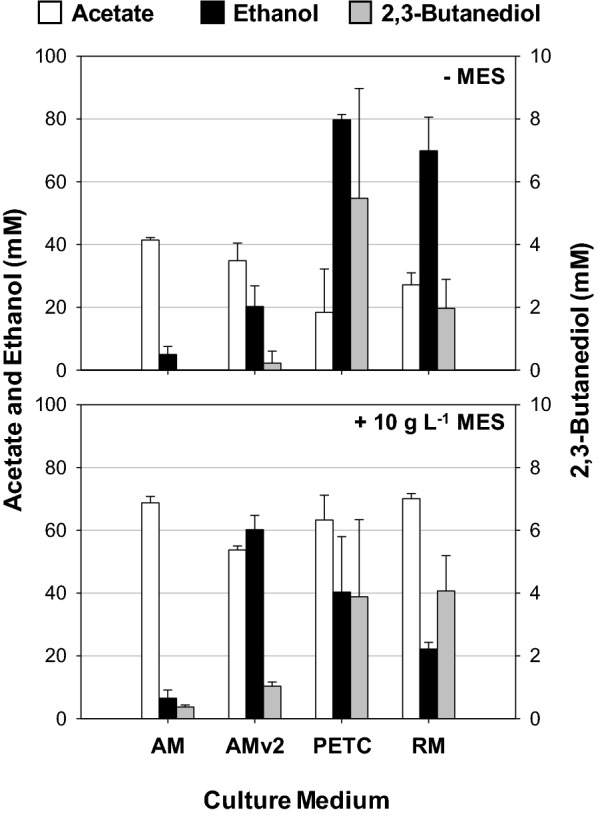
Metabolite profiles of the AWRP strain grown in various culture media with no buffering agent (top) or with the addition of 10 g L^−1^ MES (bottom). Each culture was performed in triplicate using 125-mL serum bottles, and the headspace was charged at 150 kPa with a synthetic gas mixture (50% CO, 30% N_2_, 10% CO_2_, and 10% H_2_). When MES was supplemented to the medium, final pH was adjusted to 6.0 by the addition of 1 N NaOH before sterilization

We next compared the product patterns of this bacterium grown in different culture media (Fig. 4), as the gas consumption rate and alcohol formation have been reported to be affected by medium composition [15, 26]. As medium optimization is not the principal purpose of this study, we examined metabolite profiles using four culture media with 0.5 g L^−1^ yeast extract (YE), including AM, AMv2, PETC, and RM (see “Methods” and Additional file 1: Tables S1–S3 for their compositions). AM medium was used for isolation and enrichment of the AWRP strain and is based on the DSM 614 medium. AMv2 is a modified version of AM containing increased concentrations of trace elements and phosphate. The RM medium is based on AMv2 but has much higher concentrations of trace elements than the other media, for which we primarily referred to two previous studies [15, 22]. We also cultivated *C. ljungdahlii* DSM 13528 under the same condition to determine differences between two strains.

Our results indicated that solventogenesis in strain AWRP was greatly affected by the medium composition, with ethanol titers only observed to be higher than those of acetate in PETC and RM media (Fig. 4, top). The final ethanol titer was the highest in the PETC medium (80 mM). In the RM medium, strain AWRP produced slightly less ethanol (70 mM), although the average culture time in RM was markedly shorter than in PETC (ca. 7 vs. 12 days in PETC). Small amounts of 2,3-butanediol were detected in both media as well (PETC, 5 mM; RM, 2 mM). Unexpectedly, the AWRP strain was the most poorly grown in the AM medium, even though it had been enriched in this medium: acetate was the dominant metabolite (41 mM), and only a small amount of ethanol was detected (5 mM). The effect of culture medium was also observed when using *C. ljungdahlii* (Additional file 1: Fig. S1). However, there were some differences in the product patterns. A marked difference is that *C. ljungdahlii* produced only a small amount of ethanol (3 mM) in the PETC medium and generated a higher ethanol titer than acetate in the AMv2 medium.

In addition to the unbuffered media, we tested the effect of buffering agents, since we cannot maintain the pH of culture broths at a constant value. We observed that the final pH of a culture was often below 4, which could abolish the pH gradient of the cytoplasm and cease the cellular metabolism. For a buffering agent, 2-(*N*-morpholino)ethanesulfonic acid (MES) was added to the media at a final concentration of 10 g L^−1^. The addition of MES to the culture media increased total product formation (Fig. 4, bottom), although it negatively affected the alcohol production in all media except AMv2, where ethanol production increased by threefold (60 vs. 20 mM without MES).

**Fig. 5:**
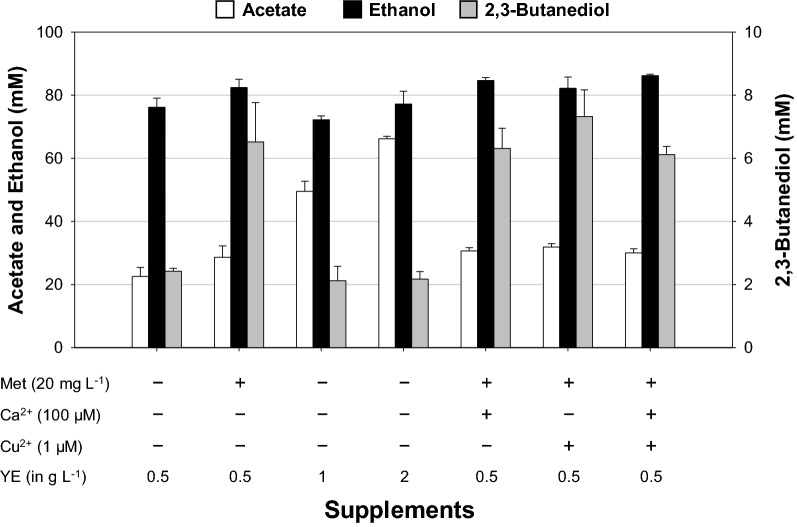
Effects of various supplements on the metabolite profiles of *Clostridium* sp. AWRP. Each culture was performed in triplicate using 125-mL serum bottles containing 10 mL of the RM medium. The headspace was charged at 150 kPa with a synthetic gas mixture (50% CO, 30% N_2_, 10% CO_2_, and 10% H_2_)

As strain AWRP showed a better CO consumption rate in the RM medium than in the PETC medium, we further examined the effects of various supplements in the RM medium, including YE, l-methionine (a potential candidate of auxotrophy identified by the genome sequence), and the metal ions that are not contained in RM but are present in PETC (Fig. 5). Interestingly, the addition of l-methionine resulted in more than a twofold increase in the 2,3-butanediol titer (6.5 vs. 2.7 mM control). Such an increase was not observed when the YE concentration was increased, which led to a subsequent increase in the acetate titer (0.5 g L^−1^, 23 mM; 1 g L^−1^, 50 mM; and 2 g L^−1^, 60 mM). The addition of Ca^2+^ or Cu^2+^ had little effect on the metabolite profiles of strain AWRP when supplemented together with l-methionine, although the addition of Cu^2+^ slightly increased the 2,3-butanediol titer.

### Metabolite profiles of strain AWRP during a bioreactor fermentation

To investigate the time course profiles for growth and metabolite production of strain AWRP, the fermentation was performed using a bioreactor. Batch fermentation was performed in a 2.5-L bioreactor containing 1.6 L of the RM medium supplemented with 0.5 g L^−1^ YE and 50 mg L^−1^ l-methionine.

**Fig. 6:**
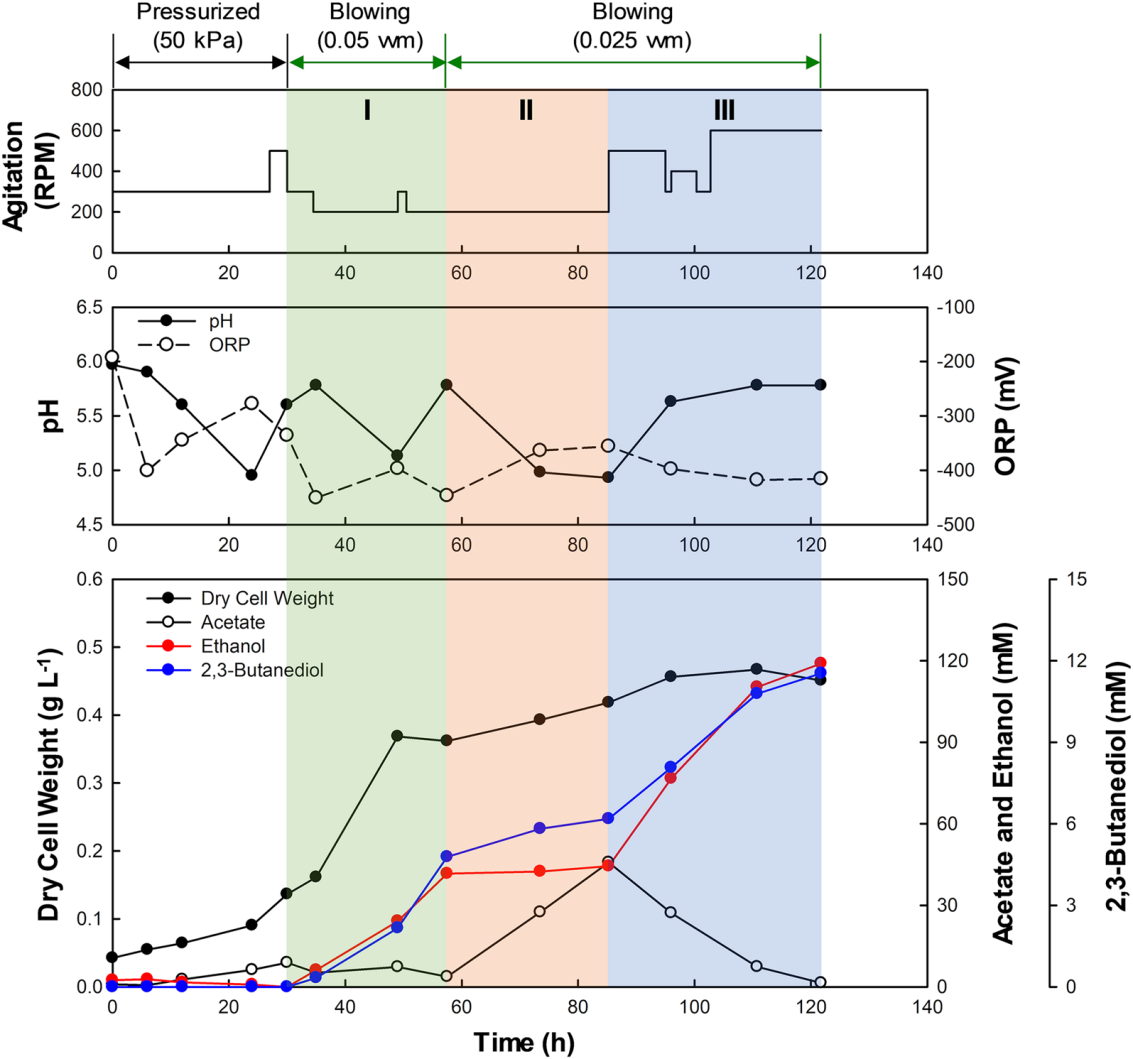
Time course profile of *Clostridium* sp. AWRP grown in a bioreactor. The gas was initially fed with pressurization of the headspace at 50 kPa until 30 h, followed by continuous flow at the ambient pressure. The pH was controlled from 35 h using ammonia (when pH ≤ 5.0) and HCl (when pH ≥ 5.8) solutions

One problem we encountered was that the growth of strain AWRP appeared to be retarded when a continuous gas supply was employed immediately after inoculation, often leading to the process failure. This was possibly due to inhibition of hydrogenases by CO, some of which are coupled to FDHs in the WL pathway [16, 27]. Thus, we performed fermentation with an initial pressurized phase (50 kPa) followed by continuous gas flow. The ORP and the metabolite profiles were significantly influenced by agitation speed and gas flow rate after continuous gas supply was applied (Fig. 6).

#### Phase I (30–57.5 h)

When continuous flow started at 30 h, the cells began to produce alcohol without notable accumulation of acetate. The specific production rate of ethanol was 191–217 mM (g DCW)^−1^ day^−1^ between 30 and 49 h, while the ethanol and 2,3-butanediol titers at 57.5 h were 42 and 5 mM, respectively. Moreover, acetate production was almost negligible during this phase (peaking at 9 mM at 30 h). The production of alcohols appeared to be coupled to a decrease in ORP. The ORP decreased to − 450 mV immediately after gas flow, and it increased again to − 400 mV after reducing agitation rate to 200 RPM. A short pulse of increased agitation (300 RPM; 49–50.5 h) also caused a decrease in ORP. The growth was not exponential, with the specific growth rate of 0.8 day^−1^ (30–35 h) that increased to 1.4 day^−1^ (35–49 h), reaching 0.37 g DCW L^−1^. This result suggested that growth inhibition by CO occurred between 30 and 35 h. Since the control of gas transfer by changing agitation speed at this flow rate was difficult, we decreased the flow rate to 0.025 vvm at 57.5 h.

#### Phase II (57.5–85.3 h)

Once the flow rate was decreased, the metabolism shifted back toward acetate production (from 4 to 46 mM), which coincided with an ORP increase to − 360 mV. The rate of acetate production was 100 mM (g DCW)^−1^ day^−1^ during this phase, which was lower than the ethanol production rate in Phase I, suggesting that gas transfer was limiting during this period. Cell mass was only slightly increased in this phase (0.4 g DCW L^−1^).

#### Phase III (85.3–121.8 h)

To determine if solvent production would reoccur by enhancing gas transfer rate, we increased agitation speed while maintaining the gas flow rate (see Fig. 6). The ORP decreased immediately after increasing the agitation rate, reaching ca. − 420 mV. In addition, the metabolism simultaneously began to shift toward alcohol production, but in contrast to Phase I, acetate assimilation contributed substantially to alcohol production. The specific production rate of ethanol was 170 mM (g DCW)^−1^ day^−1^ between 85.3 and 96 h and decreased thereafter. Increasing the agitation speed after 102.8 h affected neither the ORP nor the ethanol production rate, and final titers of ethanol, 2,3-butanediol, and acetate were 119, 12, and 1.4 mM, respectively.

## Discussion

In this study, we isolated a new alcohol-producing acetogen, *Clostridium* sp. AWRP, from the sediment of a wetland, and the fermentative characteristics of this strain were investigated with serum bottle cultures and a bioreactor experiment. The results of the 16S rRNA phylogenetic analysis indicate that this bacterium should be included in the same phylogenetic clade as *C. autoethanogenum*, *C. ljungdahlii*, *C. ragsdalei*, and *C. coskatii* (Fig. 1), which are considered as industrially important strains [5, 11, 28, 29]. Strain AWRP produces acetate, ethanol, and 2,3-butanediol from CO (see Table 2), and this strain would be the fifth gas-fermenting strain producing 2,3-butanediol. The ANI values of AWRP against the abovementioned species are below a criterion of the same prokaryotic species (Table 1) [23]. In consistent with this result, strain AWRP harbors more singletons than the others (Fig. 2b). Taken together, the AWRP strain can be reported as a new species of the genus *Clostridium*, although further investigations are necessary to clarify this issue [30].

Our results from serum bottle cultures indicated that alcohol production by strain AWRP was significantly affected by medium composition (Fig. 4). As previously demonstrated [15, 26, 31], it appears that there might be some lower limits of trace element contents as the alcohol production was the lowest in the AM medium, where the concentrations of Fe, Se, and W are the lowest among the media tested (Fig. 4). Alcohol production was highest in the unbuffered PETC medium (8–90 mM), but the RM cultures showed the shorter culture time (7 vs. 12 days PETC), possibly due to a higher gas consumption rate contributed to by higher amounts of Fe, Ni, Se, and W (see Additional file 1: Table S3), which are key elements for the metalloenzymes in the WL pathway [7, 15]. Increasing the buffering capacity of the media led to an increase in acetate accumulation in all media except for AMv2; ethanol titer was 60 mM in the MES-buffered AMv2 medium, a threefold increase when compared to the unbuffered AMv2 culture (Fig. 4). Although the reason for this result is not clear, our results should be considered when designing an experiment to optimize the culture medium for alcohol-producing acetogens, where such nontoxic but expensive buffers are widely used to relieve overacidification of the broth [15, 22, 31, 32]. The use of these buffers should be avoided for economic feasibility of a large-scale process [33].

Supplementation of several nutrients into the RM medium had little effect on alcohol production (Fig. 5). Since an analysis of the genome sequence of strain AWRP indicated that it could not synthesize l-methionine (see Fig. 3), we tested whether l-methionine supplementation would support the growth without YE, although all of our attempts were unsuccessful. This result is not surprising in acetogenic species, despite a few successful cases having been reported [11, 16]. It was previously reported that it takes several weeks for *C. autoethanogenum* and *C. ljungdahlii* to grow in a defined medium without YE [34]. A ^13^C labeling study with *C. carboxidivorans* also revealed that this bacterium has little capacity to synthesize amino acids even though its genome harbors all of the genes necessary for the synthesis of 20 proteogenic amino acids [32]. We are currently performing an adaptation experiment with gradually decreasing YE concentration in the medium with the goal of being able to grow strain AWRP in a fully defined medium. On the other hand, one unexpected observation from this study is that the l-methionine supplementation led to an increased 2,3-butanediol titer of more than twofold in presence of 0.5 g L^−1^ YE (6.5 vs. 2.7 mM Met^−^), while increasing the YE concentration only resulted in increase in the acetate titer (Fig. 5). At present, we cannot propose any possible metabolic relationship between l-methionine and 2,3-butanediol. If the methionine auxotrophy indeed exists in strain AWRP, the proteome may be affected by L-methionine concentration during growth as it is required for the translation of every protein in the cell.

It is difficult to directly compare the metabolic profile of strain AWRP with those reported for other strains in the literature due to dissimilar culture conditions (e.g., headspace pressure, gas composition, agitation, etc.). Nonetheless, it is noteworthy that strain AWRP produced larger or at least comparable amounts of ethanol in serum bottle cultures than its close relatives. In a previous study, serum bottle cultures were performed to compare the product patterns of four clostridial strains using syngas containing 50% CO and a MES-buffered PETC medium [29]. In that study, the main product was acetate in all the strains examined. The ethanol titer was the highest in *C. autoethanogenum* (40 mM), followed by *C. ragsdalei* (32 mM) [29]. More recently, *C. autoethanogenum* and *C. ljungdahlii* were cultivated in flexible gas bags containing 100% CO and a modified DSMZ 640 medium, and *C. autoethanogenum* was able to produce ca. 120 mM ethanol in ca. 39 days by acetate supplementation [35]. In the present study, AWRP produced about 40 and 63 mM of ethanol and acetate in the MES-buffered PETC medium, respectively. Moreover, the ethanol and acetate titers of 80–94 and 14–30 mM were observed in the unbuffered PETC medium (Table 2 and Fig. 4), where *C. ljungdahlii* only produced 3 mM of ethanol (Additional file 1: Fig. S1). Although our results indicate that the fermentative characteristics of strain AWRP and *C. ljungdahlii* appear to be different, there may be common factors necessary for enhancing alcohologenesis in the alcohol-producing clostridia (RM medium; see Fig. 4 and Additional file 1: Fig. S1).

The fermentation profile obtained using a batch bioreactor comes as a surprise to us. As in acetone–butanol fermentation, we presumed that acetogens would produce acids first, and then alcohols as the culture pH decreases [12, 36–38]. However, alcohol production by strain AWRP appears not to require either acetate accumulation or a low pH value. When continuous gas flow began, the pH and the acetate titer were 5.6 and 9 mM, respectively (30 h; Fig. 6). This result clearly distinguished strain AWRP from the fermentation of *C. carboxidivorans*, which shows typical metabolic shift from acidogenesis to alcohologenesis [12]. More recently, chemostat experiments were performed using a proprietary mutant of *C. autoethanogenum* to investigate the effects of gas compositions on the metabolic profiles [20]. Although a high titer of ethanol was achieved (~ 260 mM) when grown on a mixture of CO-H_2_, the ethanol production of the strain appeared to be more dependent on H_2_ rather than CO [20]. In strain AWRP, alcohols were only produced when the ORP was below − 400 mV (Fig. 6), at which point hydrogen was hardly consumed due to CO inhibition. It was reported that the FDH activity was also inhibited by CO in a hydrogenase–FDH complex, in addition to the hydrogenase activity [16]. If a similar inhibition exists in strain AWRP, it is likely that the CODH genes other than *acsA* may be involved in CO oxidation to produce reduced ferredoxin, which Rnf complex and AOR can use to produce ATP and ethanol, respectively. Further investigation into the physiology of strain AWRP and its close relatives will yield insights into alcohologenesis and the underlying genetic regulations in acetogenic clostridia [19, 20, 39, 40].

In the bioreactor experiment, the specific ethanol production rates of AWRP in Phases I and III were 134–217 and 41–172 mM (g DCW)^−1^ day^−1^, respectively. In addition, the residual acetate titer was only 1 mM at the end of fermentation, and the final ethanol/acetate ratio was more than 70 (Fig. 6). To the best of our knowledge, such a high alcohol selectivity has not been reported in other clostridial species. Homoethanol production has been demonstrated in *C. autoethanogenum* by optimizing the culture medium, but the final ethanol titer was 20 mM at most [26]. In another study where *C. autoethanogenum* was used for a chemostat experiment, specific production rates of ethanol and acetate of 126 and 62 mM (g DCW)^−1^ day^−1^ were observed, respectively, when operated with a dilution rate of 1.8 day^−1^ [16]. Our results suggest that strain AWRP can also be a prominent host for alcohol production from syngas; the low cell density (~ 0.5 g L^−1^) and the auxotrophy should be solved to increase the volumetric CO consumption rate and alcohol productivity. As the genetic modifications on acetogenic bacteria are now possible, further genetic and metabolic engineering studies will answer those issues aforementioned [14, 21, 22, 41].

## Conclusions

This study describes the isolation of a novel acetogen *Clostridium* sp. AWRP, which produces acetate, ethanol, and 2,3-butanediol from syngas. From the complete genome of strain AWRP, it was observed that this strain may be a novel species of the genus *Clostridium*. Strain AWRP produced considerable amounts of ethanol in serum bottle cultures, which were higher than or comparable to typical values obtained using other acetogenic clostridia. Moreover, the results of our bioreactor experiment showed that the alcohol production of strain AWRP might be neither acetate- nor low pH- but ORP-driven, which has not been reported previously in its close relatives. The elucidation of the complete genome of strain AWRP will make it possible to conduct comparative studies of alcohol-producing acetogens in the future. We anticipate that this bacterium will be useful for studying not only alcohol production mechanisms in acetogenic clostridia but also enhanced alcohol production from syngas through systems metabolic engineering.

## Methods

Unless otherwise noted, pressure values are expressed as gauge pressure.

### Isolation procedure

For enrichment and isolation of autotrophic microorganisms, the following basal medium (AM) was used, which contained per liter (in grams unless indicated otherwise): NH_4_Cl, 1.0; K_2_HPO_4_, 0.33; MgCl_2_, 0.52; CaCl_2_·2H_2_O, 0.1; KCl, 0.33; NaCl, 1.0; NaHCO_3_, 1.0; l-cysteine-HCl, 0.5; Bacto™ yeast extract, 0.5; trace element solution (DSM 141), 10 mL; 0.1% resazurin solution, 1 mL. After adjusting the pH to 5.5 using 1 N HCl, the medium was dispensed into Wheaton serum vials, which were then purged with N_2_ and sterilized by autoclaving. Finally, sterile Na_2_S·9H_2_O solution (to a final concentration of 0.05 g L^−1^) and 100× Wolfe’s vitamin solution were added to the medium (see Ref. [42] for the composition of the vitamin solution). Sediments and livestock sludge samples were collected from marine sediments, rice paddies, lakes, wetlands, poultry and cattle farms. The samples were stored in sterile sampling bags and brought into an anaerobic chamber (Coy Laboratory Products, MI, USA) containing 95% N_2_ and 5% H_2_. Samples were transferred to 50-mL serum vials containing 20 mL of the AM medium, and each slurry was used as an inoculum at two different dilutions (10^−1^ and 10^−2^), each in duplicate. Enrichment culture was carried out at 30 °C for 2 weeks in 25-mL serum vials containing 5 mL of the AM medium. The headspace gas was exchanged immediately after inoculation with a synthetic blend gas that mimicked a composition of synthesis gas (50% CO, 10% H_2_, 10% CO_2_, and 30% N_2_; 150 kPa). Whenever positive cultures were identified by an increase in turbidity, they were transferred to fresh medium (10% inoculum). The enriched samples were streaked onto AM agar plates, which were then stored and incubated in an anaerobic jar containing 150 kPa of the syngas.

### Genome sequencing

For genome sequencing, RNA-free genomic DNA was extracted using the cetyltrimethylammonium bromide (CTAB) method [43]. The closed genome sequence of *Clostridium* sp. AWRP was determined by PacBio single-molecule real-time (SMRT) sequencing at DNALink (Seoul, Korea). A SMRTbell template preparation kit 1.0 (Pacific Biosciences, CA, USA) was used according to the PacBio standard protocol. Small fragments (< 20 kb) were excluded using a BluePippin size selection system (Sage Science, Inc., MA, USA) to construct a large-insert library. The SMRT cells were run on a PacBio RSII instrument (Pacific Biosciences, CA, USA) using a P6-C4 chemistry combination. De novo assembly was performed using the Hierarchical Genome Assembly Process (HGAP, version 2.3), which included consensus polishing with Quiver [44]. The complete genomes were annotated using automatic annotation pipeline in GenBank. The genome sequence of strain AWRP has been deposited in GenBank (http://www.ncbi.nlm.nih.gov/GenBank/) under accession number CP029758.

### Bioinformatic analyses

For the construction of a phylogenetic tree, the consensus sequence of all the 16S ribosomal RNA genes of strain AWRP was used. The 16S rRNA sequences of other species were obtained from the NCBI database. These sequences were aligned using ClustalX [45], and the resulting alignment was then used to calculate an evolutionary distance matrix according to the F84 model [46] using the PHYLIP 3.695 package [47]. The phylogenetic tree was inferred by the neighbor-joining method [48]. The robustness of the neighbor-joining tree topology was validated using bootstrap analysis with 1000 replicates [49]. The ETE 3 Python package was employed for visualization of the phylogenetic tree [50]. Orthologous gene analysis was performed using Proteinortho 6.0b [51], with the same parameters described in a previous study [29].

### Anaerobic cultivation

*Clostridium ljungdahlii* DSM 13528 was purchased from German Collection of Microorganisms and Cell Cultures (DSMZ). For routine propagation, *Clostridium* sp. AWRP and *C. ljungdahlii* were grown in LBFA medium. The LBFA medium was composed of (in g L^−1^): Bacto™ yeast extract, 5; Bacto™ tryptone, 10; NaCl, 0.5; fructose, 5; CH_3_COONa·3H_2_O, 5; agar, 15 (as needed).

To characterize the metabolite profile of strain AWRP in serum bottles, four cultivation media were employed: AM, AMv2, modified PETC, and RM (see “Isolation procedure” section and Additional file 1: Tables S1–S3). Unless otherwise noted, all media were supplemented with 0.5 g L^−1^ of yeast extract. When needed, 10 g L^−1^ MES was supplemented to the culture broth and the final pH was adjusted to 6.0 using 1 N NaOH solution before sterilization. An active AWRP culture grown in LBFA was used to inoculate a seed culture (5% inoculum) in a 125-mL serum bottle (actual average volume of ca. 160 mL) containing 20 mL of medium and 50 kPa of the syngas in the headspace. The active seed culture was transferred (10% inoculum) to three 125-mL serum vials containing the same medium and 150 kPa of the syngas. The serum bottles were incubated at 37 °C and 180 RPM on a rotary shaker.

### Bioreactor experiments

A 2.5-L bioreactor (BioCNS, Daejeon, Korea) was used in this study, which was equipped with three baffles, three six-blade Rushton turbines, a microsparger, and a mass flow controller as described previously [52, 53]. A 2.5-L steel gas reservoir was connected between the condenser of the bioreactor and a wet gas meter (W-NK-0.5; Shinagawa Co. Ltd., Tokyo, Japan) for the initial pressurization phase. Integrated probes were used to monitor pH and ORP in the broth. Antifoam 204 (Sigma-Aldrich, St. Louis, MO) was added manually to control foaming during operation, and pH was maintained between 5.0 and 5.8 by the automatic addition of 5 N NH_4_OH and 2 N HCl solutions, where no pH control performed within this range.

Seed cultures were prepared by inoculating the LBFA-grown culture at 37 °C in four 125-mL serum bottles, each containing 40 mL of RM medium (supplemented with 0.5 g L^−1^ YE and 20 mg L^−1^ l-methionine). The bottles were initially fed 100 kPa of a gas mixture (20% CO_2_ and 80% H_2_) for 24 h, after which the headspaces were replaced with 100 kPa of the syngas mixture. Whenever the headspace CO concentration decreased below 25 mM, the atmosphere was flushed and pressurized at 100 kPa with the same syngas mixture. The seed culture (OD_600_ ~ 2.0) was used to inoculate the bioreactor containing 1.44 L of RM medium supplemented with 0.5 g L^−1^ yeast extract and 50 mg L^−1^ l-methionine. After sterilization, the headspace of the bioreactor was sufficiently flushed with pure-grade N_2_ (99.999%) at a flow rate of 200 mL min^−1^ for at least 2 h. The syngas mixture was supplied at a flow rate of 200 mL min^−1^ for 1 h before inoculation. At this step, the actual flow rate was double confirmed by measuring the off-gas flow rate with a wet gas meter (W-NK-0.5; Shinagawa Co. Ltd., Tokyo, Japan), and phosphate and vitamin solutions were added. After the headspace was pressurized at 50 kPa, a reducing agent solution (100× conc.; see Additional file 1: Table S3) was added to the medium.

### Analytical methods

Cell density was determined by measuring the OD_600_ using a spectrophotometer (Biophotometer Plus; Eppendorf, Hamburg, Germany). The dry cell weights were calculated from the following correlation: 0.27 g DCW (OD_600_)^−1^, which was determined from the samples taken from RM cultures. Headspace gas samples were taken using a gastight syringe (1710; Hamilton Company, NV) and analyzed using a gas chromatograph (YL 6100; YL Instrument Co., Anyang, South Korea). The GC was equipped with a Porapak N (45/60 mesh, 10 ft. × 1/8 in., Supelco; for separation of CO_2_ from other species) and a 13X molecular sieve (3 ft. × 1/8 in., Supelco; for separation of H_2_, N_2_, and CO) column. The GC was also equipped with a thermal conductivity detector (for detection of H_2_ and N_2_) and a flame ionizing detector in combination with a methanizer (for detection of CO and CO_2_). The outlet of the Porapak N and the inlet of the 13X column were connected through a six-port switching valve (Valco Instruments, Houston, TX) to make CO_2_ bypass the 13X column after analysis of CO and H_2_. Argon was used as the carrier gas at a flow rate of 30 mL min^−1^. The metabolite concentrations in the culture broth were determined using an HPLC-RID system (YL 9100; YL Instrument Co.) equipped with an Aminex HPX-87H (300 × 7.8 mm; Bio-Rad, CA) column with a sulfuric acid solution (5 mM) used as the mobile phase. Ethanol and 2,3-butanediol concentrations were double confirmed using a GC-FID system (Scion 456; Scion Instruments, Livingston, United Kingdom) equipped with an DB-Wax capillary column (30 m length × 0.53 μm ID × 1 μm thickness; Agilent, Santa Clara, CA).

## Supplementary information

**Additional file 1: Fig. S1.** Product patterns of *C. ljungdahlii* DSM 13528 grown on various culture media (unbuffered); **Table S1.** Composition of the AMv2 medium; **Table S2.** Composition of the modified PETC medium; **Table S3.** Composition of the RM medium.

## Data Availability

*Clostridium* sp. AWRP has been deposited in the Korean Collection for Type Cultures (KCTC) with the accession number of KCTC 13908BP.

## Abbreviations

AOR: Aldehyde:ferredoxin oxidoreductase
CODH: Carbon monoxide dehydrogenase
FDH: Formate dehydrogenase
gANI: Genome-wide average nucleotide identity
MES: 2-(*N*-morpholino)-ethanesulfonic acid
Met: l-methionine
ORP: Oxidoreduction potential
YE: Yeast extract
