# Supplementary material for: Domestication of the novel alcohologenic acetogen Clostridium sp. AWRP: from isolation to characterization for syngas fermentation
Source: Biotechnol Biofuels. 2019 Sep 23;12:228. doi: 10.1186/s13068-019-1570-0 (PMC6757427; doi:10.1186/s13068-019-1570-0)
Supplement: Supplementary file 1 — Additional file 1: Fig. S1. Product patterns of C. ljungdahlii DSM 13528 grown on various culture media (unbuffered); Table S1. Composition of the AMv2 medium; Table S2. Composition of the modified PETC medium; Table S3. Composition of the RM medium. [file 13068_2019_1570_MOESM1_ESM.docx]

**Additional Information for**

**Domestication of the Novel Alcohologenic Acetogen *Clostridium* sp. AWRP: From Isolation to Characterization for Syngas Fermentation**

Joungmin Lee^1^, Jin Woo Lee^1^, Cheol Gi Chae^1,2^, Soo Jae Kwon^1,2^, Yun Jae Kim^1,2^, Jung-Hyun Lee^1,2^, and Hyun Sook Lee^1,2,*^

^1^Marine Biotechnology Research Center, Korea Institute of Ocean Science and Technology, Haeyangro 385, 49111 Busan, Republic of Korea

^2^Department of Marine Biotechnology, University of Science and Technology, Daejeon, Republic of Korea.


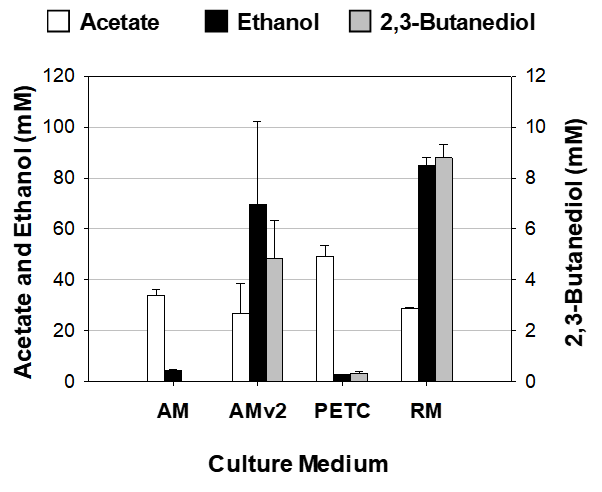


**Fig. S1** Product patterns of *C. ljungdahlii* DSM 13528 grown on various culture media (unbuffered).

**Table S1** Composition of the AMv2 medium*^a^*

| Component | Concentration (amount per L) |
| --- | --- |
| KH_2_PO_4_ | 0.5 g |
| K_2_HPO_4_ | 0.5 g |
| MgSO_4_·7H_2_O | 0.2 g |
| CaCl_2_·2H_2_O | 0.1 g |
| NaCl | 1 g |
| NH_4_Cl | 1 g |
| L-Cysteine·HCl | 0.3 g |
| Yeast extract | 0.5 g |
| Trace element solution I*^b^* | 1 mL |
| Trace element solution II*^c^* | 1 mL |
| Wolfe’s vitamin solution*^d^* | 10 mL |
| 3% Na_2_S·9H_2_O | 10 mL |

*^a^*Once all ingredients except vitamins and Na_2_S were dissolved, the pH was adjusted to 6.0 with 1 N NaOH solution. Vitamins and Na_2_S were added sterilization.

*^b^*One liter of the trace element solution I contained the following ingredients (amount in grams unless otherwise noted): 35% HCl, 10 mL; FeSO_4_·7H_2_O, 13.9; MnCl_2_·4H_2_O, 9.9; ZnCl_2_, 1.36; CoCl_2_·6H_2_O, 2.38; CuCl_2_·2H_2_O, 0.34; NiCl_2_·6H_2_O, 0.48

*^c^*One liter of the trace element solution II contained the following ingredients (amount in grams): Na_2_MoO_4_·2H_2_O, 1.65; Na_2_SeO_3_·5H_2_O, 0.86; Na_2_WO_4_·2H_2_O, 1.65; NaOH, 0.4

*^d^*One liter of the solution contained the following ingredients (amount in milligrams): biotin, 2; folic acid, 2; pyridoxine·HCl, 10; thiamine·HCl, 5; riboflavin, 5; nicotinic acid, 5; Calcium D-pantothenate, 5; vitamin B12, 0.1; *p*-aminobenzoic acid, 5; lipoic acid, 5

**Table S2** Composition of the modified PETC medium*^a^*

| Component | Concentration (amount per L) |
| --- | --- |
| KH_2_PO_4_ | 0.1 g |
| MgSO_4_·7H_2_O | 0.2 g |
| CaCl_2_·2H_2_O | 0.02 g |
| NaCl | 0.8 g |
| KCl | 0.1 g |
| NH_4_Cl | 1.0 g |
| Yeast extract | 0.5 g |
| Trace element solution*^b^* (ATCC 1754) | 10 mL |
| Wolfe’s vitamin solution | 10 mL |
| Reducing agent*^c^* | 10 mL |

*^a^*Once all ingredients except vitamins and reducing agents were dissolved, the pH was adjusted to 5.9 with 1 N NaOH solution. Vitamins and reducing agents were added after sterilization.

*^b^*One liter of the trace element solution contained the following ingredients (amount in grams): nitrilotriacetic acid, 2; MnSO_4_·H_2_O, 1; Fe(SO_4_)_2_(NH_4_)_2_·6H_2_O, 0.8; CoCl_2_·6H_2_O, 0.2; ZnSO_4_·7H_2_O, 0.0002; CuCl_2_·2H_2_O, 0.00002; NiCl_2_·6H_2_O, 0.00002; Na_2_MoO_4_·2H_2_O, 0.00002; Na_2_SeO_4_, 0.00002; Na_2_WO_4_, 0.00002

*^c^*One liter of the reducing agent contained the following ingredients (amount in grams): NaOH, 9; L-Cysteine·HCl, 40; Na_2_S·9H_2_O, 40

**Table S3** Composition of the RM medium*^a^*

| Component | Concentration (amount per L) |
| --- | --- |
| MgSO_4_·7H_2_O | 0.2 g |
| NaCl | 1 g |
| KCl | 0.2 g |
| NH_4_Cl | 1 g |
| Yeast extract | 0.5 g |
| Phosphate solution*^b^* | 10 mL |
| Trace element solution I*^c^* | 1 mL |
| Trace element solution II*^d^* | 1 mL |
| Wolfe’s vitamin solution | 10 mL |
| Reducing agent*^e^* | 10 mL |

*^a^*Vitamins, phosphate, and reducing agents were added after sterilization. The pH of the medium was not adjusted.

*^b^*One liter of the phosphate solution contained the following ingredients (amount in grams): NaH_2_PO_4_·2H_2_O, 80; K_2_HPO_4_, 40

*^c^*One liter of the trace element solution I contained the following ingredients (amount in grams unless otherwise noted): 35% HCl, 10 mL; FeSO_4_·7H_2_O, 27.8; MnCl_2_·4H_2_O, 9.9; ZnCl_2_, 4.09; CoCl_2_·6H_2_O, 11.9; NiCl_2_·6H_2_O, 2.38

*^d^*One liter of the trace element solution II contained the following ingredients (amount in grams): Na_2_MoO_4_·2H_2_O, 0.24; Na_2_SeO_3_·5H_2_O, 1.32; Na_2_WO_4_·2H_2_O, 3.3

*^e^*One liter of the reducing agent contained the following ingredients (amount in grams): NaOH, 7.61; L-Cysteine·HCl, 30; Na_2_S·9H_2_O, 30
